# Complex Geometry Cellulose Hydrogels Using a Direct Casting Method

**DOI:** 10.3390/bioengineering7020058

**Published:** 2020-06-16

**Authors:** Hossein Najaf Zadeh, Tim Huber, Volker Nock, Conan Fee, Don Clucas

**Affiliations:** 1 Department of Mechanical Engineering, University of Canterbury, Private Bag 4800, Christchurch 8020, New Zealand; don.clucas@canterbury.ac.nz; 2 Biomolecular Interaction Centre, University of Canterbury, Private Bag 4800, Christchurch 8020, New Zealand; tim.huber@canterbury.ac.nz (T.H.); volker.nock@canterbury.ac.nz (V.N.); conan.fee@canterbury.ac.nz (C.F.); 3 School of Product Design, University of Canterbury, Private Bag 4800, Christchurch 8020, New Zealand; 4 Department of Electrical and Computer Engineering, University of Canterbury, Private Bag 4800, Christchurch 8020, New Zealand

**Keywords:** cellulose, hydrogel, physical cross-linking, investment casting, wax mould

## Abstract

To facilitate functional hydrogel part production using the indirect wax mould method, it is necessary to understand the relationships between materials, process and mould removal. This research investigated the thermophysical properties, wettability and surface roughness of wax template moulds in the production of cellulose hydrogel objects. Cellulose gel was thermally formed and shaped in three different wax moulds—high melting point paraffin, sacrificial investment casting wax and Solidscape^®^ wax—by physical cross-linking of polymer networks of cellulose solution in NaOH/urea aqueous solvent. All three wax moulds were capable of casting cellulose hydrogel objects. Cellulose gelling time was reduced by increasing the temperature. Thus, the mould melting temperature had a direct effect on the gelling time. It was found that mould removal time varied based on the contact angle (CA) of the cellulose solution and the mould, and based on the melting point of the mould. A higher CA of cellulose solution on the wax moulds resulted in faster mould removal. When melting the wax in 90 °C water, high melting point paraffin, sacrificial investment casting and Solidscape^®^ wax took about 3, 2 and 1.5 h, respectively, to remove the moulds from the cellulose gel.

## 1. Introduction

Hydrogels are three-dimensional (3D) polymer networks that can absorb and retain significant amounts of water [1], forming versatile materials with various applications including tissue engineering [2,3], drug delivery [4], food and agriculture [5], purification and sensors [6]. The fabrication of hydrogels from various natural polymers such as starch, gelatine, alginate, chitosan and cellulose has gained attention due to their potential application in the biomaterials field [7]. Cellulose is a renewable and biodegradable polysaccharide found in wood and plants, which has an amphiphilic structure with polar (OH) and nonpolar (CH) groups [8,9]. Cellulose contains abundant hydroxyl groups, which are essential for hydrogel formation [7]; however, the supramolecular architecture—specifically, the strong cellulose–cellulose hydrogen bonds—makes it insoluble in water after gelling [10,11]. Therefore, cellulose dissolution results from a solvent’s ability to eliminate the inter- and intramolecular hydrogen bonds among biopolymer molecules [12]. It has been reported that cellulose can be dissolved in an aqueous solvent of NaOH/urea, to create a solution which forms an irreversible gel in response to cooling or heating [13,14]. However, cellulose gel has a weak mechanical structure and is very difficult to shape [15]. To this end, methods such as dry–wet spinning [16] and sacrificial templating have been used to shape cellulose hydrogel [17,18,19]; recently, three-dimensional (3D) printing of cellulose gel using a 3D bioprinter has been used to shape cellulose hydrogel [20]. 

Additive manufacturing (AM) can be employed to shape hydrogels. The method is sufficiently versatile to enable the fabrication of complicated geometries. Two-photon lithography [21] and nozzle extrusion bioprinting [22] are two examples of AM techniques that are commonly used for the fabrication of tissue-engineered scaffolds. However, these approaches are suitable for only a limited range of polymers [23], for example, thermosensitive polymers in the case of extrusion techniques [24] and photopolymerizable resins in the case of photolithography [25]. As a result, these methods have limited utility in the fabrication of self-assembling hydrogels. To overcome these obstacles, inverse sacrificial templating—an indirect wax mould method—can be used to produce porous hydrogels [17,19,26,27]. This method enables chemical modification and enhancement of the mechanical properties of the hydrogels produced, as well as the high-speed fabrication of large complex geometries, such as triply periodic minimal surfaces (TPMSs) [28]. AM methods such as thermojet technology can be used for the fabrication of moulds. This technique can produce wax models by jetting tiny droplets (5000 dots per inch) of melted liquid material, which cool and harden on impact to form the solid object [29]. An alternative method uses dissolvable sacrificial 3D printed master moulds made of materials such as polyvinyl alcohol PVA [17] and acrylonitrile butadiene styrene (ABS) [30]. The wax casting method can be applied to a range of hydrogel materials. We studied the fabrication of renewable, high-aspect-ratio cellulose hydrogel using the proposed method, which employs a wax mould as a thermoplastic sacrificial template material. 

Wax is a suitable material for a sacrificial templating process to fabricate cellulose hydrogel [31]. It is non-toxic, hydrophobic and insoluble in water. In addition, it can be recycled and reused when employed as a mould in our proposed method. During the casting process, the cellulose solution should wet the internal surfaces of the wax mould to create fine features (<50 micrometres) and avoid the formation of cavities before the solution forms an irreversible gel. A lower contact angle (CA) between the wax mould and the cellulose solution indicates better wettability of the wax by the cellulose solution—a desirable property. The mould also needs to withstand the heat required to induce gelation. It should not interact with the cellulose solution and should be easily removable after the cellulose has gelled, leaving no residue on the cast cellulose hydrogel. 

Cellulose gel forms through heat-induced physical cross-linking due to the strong self-association tendency of cellulose chains [32]. If the heating temperature reaches the wax mould’s melting point, the casting wax will start to melt, affecting the shape of the cellulose gel. Therefore, the heating temperature must allow the cellulose to reach its gelling point without melting the wax mould. The present study investigated three types of waxes as potential mould materials for cellulose hydrogel investment casting as follows. (i) High melting point paraffin, hereafter referred to as wax 1; although not typically used in its pure form for mould making, this is a well-known wax with many industrial applications [33]. (ii) Sacrificial investment casting wax, or lost-wax, hereafter referred to as wax 2, has been used for decades to cast metal parts and as an economical material for batch manufacturing [34]. (iii) Solidscape^®^, hereafter referred to as wax 3, is a wax specifically developed for use as a support material in a thermojet 3D printer, with applications in medicine [35,36], dentistry [35,36,37,38] and tissue engineering [39]. The different thermophysical properties of the selected waxes can affect the speed of the hydrogel fabrication process, including gelling time and mould removal time, as well as the shape of the resulting parts, including the formation of defects. We investigated the interactions between these three waxes as mould materials and the cellulose solution used to create the hydrogel by measuring the CA to evaluate surface wetting. We also studied the thermophysical behaviours of the mould materials to determine the effect of wax melting temperature on the mould removal process. The effects of mould removal on mechanical properties, porosity and shrinkage of the fabricated cellulose hydrogels were investigated in our previous work [30]. It was found that mould materials and removal methods have a significant effect on the mechanical properties and microstructure of cellulose hydrogel. Larger pore sizes decreased the compression strength and modulus of cellulose hydrogel samples. A balance between the porosity and density for a cellulose hydrogel part must be established for the specific applications.

## 2. Materials and Methods 

### 2.1. Materials

High melting point paraffin (melting point > 56 °C) and urea (ACS grade) were purchased from Sigma-Aldrich (St. Louis, MO, USA). Sodium hydroxide was purchased from Thermo Fisher Scientific (Waltham, MA, USA.) The cellulose used was Sigmacell cellulose powder, Type 20, also purchased from Sigma-Aldrich. Solidscape^®^ wax (support material for Solidscape^®^ 3D Printers) and sacrificial wax (Pink wax) were purchased from Regal Casting (Auckland, New Zealand). The probe liquid used for CA measurements was 5 wt. % cellulose solution. All chemicals were used as received.

To prepare the cellulose solvent, 12 wt. % urea and 7 wt. % NaOH were dissolved in deionised water, then cooled to 4 °C; subsequently, 5 wt. % cellulose powder (Sigmacell, Sigma-Aldrich) was dispersed in the solvent using a Silverson overhead mixer (Silverson Machines, Inc., East Longmeadow, MA, USA) at 5000 rpm for approximately 3 min. The solution was kept at −12 °C for 4–6 h to achieve a clear solution and then stored at 1–2 °C until use, with a maximum storage time of 3 days to avoid degradation.

For CA analysis, cellulose gel specimens were prepared by pouring 5 wt. % cellulose solution into a Perspex mould (40 mm × 20 mm × 5 mm) and heating to 80 °C for 5 min in an oven to induce gelation. 

For the preparation of wax specimens, microscope slides were cleaned by washing with water and detergent, then with 72% ethanol, followed by rinsing with deionised water for 5 min and acetone for a further 5 min. The slides were left to dry for 30 min. Then, they were dipped into the melted wax and cooled to room temperature. Slides were triple coated with the waxes. The wax-coated slides were used for wettability investigation of cellulose solution and wax materials.

### 2.2. Methods

**Figure 1:**
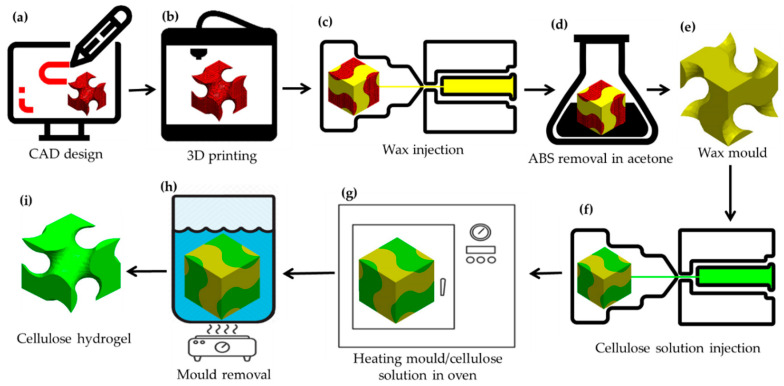
Steps in the fabrication of structured porous cellulose hydrogel: (**a**) CAD design of network gyroid with 1-mm channel size, (**b**) gyroid mould three-dimensional (3D) printed out of acrylonitrile butadiene styrene (ABS) using Up-box mini 2 3D printer, (**c**) wax injected into the 3D printed ABS, (**d**) ABS and wax mould in acetone solvent, (**e**) resulting wax mould after removal of ABS, (**f**) cellulose solution injected into the wax mould, (**g**) heating moulds to gel cellulose solution in an oven, (**h**) mould removal in 90 °C water and (**i**) final cellulose hydrogel.

A gyroid part as a TPMS with a 1-mm channel size was designed as a sacrificial template. A Schoen gyroid unit cell was created using Mathematica (12.1, Wolfram Research, Champaign, Illinois, USA) and patterned in x-, y- and z-axes using Solidworks (2018, Dassault Group, Vélizy-Villacoublay, France) to create a network gyroid pattern (Figure 1a). The model was saved as an STL format and 3D printed out of acrylonitrile butadiene styrene (ABS) using a fused deposition modelling (FDM) UpBox mini2 3D printer (Tiertime, Beijing, China) (Figure 1b). Waxes 1, 2 and 3, maintained at molten phase above their melting points, were poured separately into the ABS moulds and cooled to ambient temperature (24 ± 2 °C, 55% relative humidity) to solidify (Figure 1c). The parts were then placed into acetone for 24 h to remove the 3D-printed ABS plastic (Figure 1d) and leave the negative wax moulds (Figure 1e). Following this, cellulose solution was injected into the negative wax moulds (Figure 1f) and gelled by heating at set temperatures of 57 °C, 47 °C and 41 °C for waxes 1, 2 and 3, respectively (Figure 1g). The wax moulds with the cellulose gel were immersed in 90 °C water (above the melting point of the wax) for mould removal (Figure 1h), then the cellulose gel was immersed in distilled water and regenerated by replacing the solvent with water (Figure 1i). 

In the gelation process, the moulds filled with cellulose solution were heated to reach the gelling point. Higher heating temperatures resulted in faster gelling time. However, there was a limit for setting the peak temperature. The peak temperature should be set based on the onset of the melting point of the mould materials. By the completion of the gelling process, the wax moulds should be removed. The interaction of melted wax and cellulose gel was studied by placing melted wax at 70 °C on cellulose gel. This was observed using commercial contact-angle analyser software and a goniometer (KSV, CAM200, Helsinki, Finland) equipped with a Nikon (Tokyo, Japan) digital camera.

To investigate the completion of mould removal, we calculated the weight of the cellulose powder in the cellulose solution injected into each mould, and compared this to the dry weight of freeze-dried cellulose hydrogel after mould removal. For each wax material (1, 2 and 3), 10 samples were made. Every 30 min from the starting point of wax removal, over the course of 5 h, one sample was removed from the hot water. 

The static CA of liquid droplets on test specimens was measured using the commercial contact-angle analyser software and the goniometer apparatus. A micro-syringe was used to deposit volume-controlled droplets onto the test specimens, including both the wax and cellulose gel surfaces. All experiments and measurements were performed under ambient laboratory conditions of 24 ± 2 °C and 55% relative humidity and repeated 10 times for each wax. 

The CA was calculated using the software of the goniometer to model the mechanical equilibrium of a drop under three interfacial tension actions: liquid–vapour, solid–vapour and solid–liquid, using the Young–Laplace equation [40,41]:

(1) $$\gamma_{lv}\;cos\;\theta_{Y}=\gamma_{sv}-\gamma_{sl}$$

where $\gamma_{lv},\;\gamma_{sv},\;\mathrm{and}\;\gamma_{sl}$ are liquid–vapour, solid–vapour and solid–liquid interfacial tensions, respectively, and $\theta_{Y}$ is the CA (also known as Young’s contact angle) [42,43]. 

To enable reproducibility and repeatability of the CA measurement in the present study, the technique of recently advanced contact angle was used. Droplets of liquid were sequentially deposited on a surface until a base diameter of 5–7 mm was reached [42]. The goniometer was modified to enable deposition of heated liquids onto a surface; the heating system was a custom-controlled temperature device that used a microcontroller. This system was used to deposit melted wax onto the cellulose gel substrate, enabling precise control of the temperature of the melted wax and the deposition volume.

The thermal properties of the waxes, such as their melting and solidification temperatures and latent heats of fusion, were measured using the differential scanning calorimetry (DSC) technique (DSC8500; PerkinElmer, Waltham, MA, USA). Wax samples of ~7 mg were sealed in an aluminium pan. The analyses were performed in the temperature range of 0–95 °C with heating rates of 1 and 10 °C min^−1^, followed by cooling rates of 50, 25 and 10 °C min^−1^ (Table S1). A constant nitrogen stream was supplied at a volume flow rate of 20 mL min^−1^ throughout the tests. Samples were prepared by being subjected to a heating and cooling cycle prior to the test to eliminate any thermal memory effects. To calculate the melting point of the waxes, a tangent line was automatically drawn using Pyris analysis software (13.3.2.0030, PerkinElmer, Waltham, MA, USA) at the largest slope on the face of the selected graph. The latent heat of fusion was calculated using the area under the peak of the DSC curve [44]. 

Phase and height images were acquired using a Nanoscope atomic force microscope (AFM) (Digital Instruments Dimension 3100, Santa Barbara, CA, USA). Measurements were taking in tapping mode with scan rates between 0.5 and 1.0 Hz. The standard lateral and vertical deviations of the AFM measurements were varied based on the sample to account for uneven surfaces on the wax samples and the aspect ratio of the AFM tip. Mean roughness values (Ra), which represent the arithmetic average of the deviation from the centre plane, were measured from the AFM images using Gwyddion software (V.2.52, www.gwyddion.net) [45] in height mode over 50 µm × 50 µm image dimensions.

## 3. Results

### 3.1. Mould Wettability

**Figure 2:**
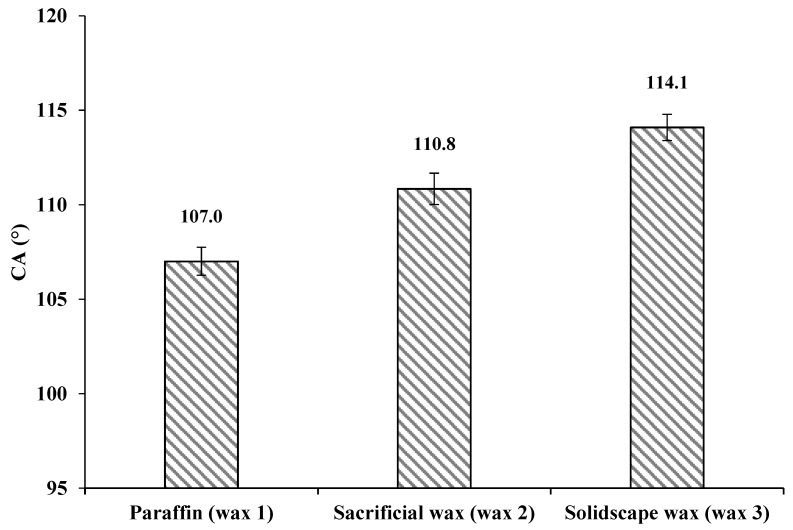
Contact angles of cellulose solution on paraffin (wax 1) 107° ± 0.74°, sacrificial investment casting wax (wax 2) 110.8° ± 0.83° and Solidscape^®^ wax (wax 3) 114.1° ± 0.7°. Abbreviations: CA, contact angle.

We determined the contact angles of cellulose solution to be 107° ± 0.74°, 110.8° ± 0.83° and 114.1° ± 0.7° on waxes 1, 2 and 3, respectively (Figure 2). Similar values have been reported for wax 1 with water as a liquid droplet by Zisman (θ = 105–110°) [46], Ström (θ = 103–108.5°) [47], Wenzel (θ = 109–112°) [48] and Kamusewitz (θ = 103°) [49].

In general, contact angles of 90° < θ < 180° do not cause surface wetting, and the solid material can thus be considered hydrophobic. Contact angles of 0° < θ < 90° cause wetting and indicate a hydrophilic solid [42]. A flat surface was found to be essential for accurate measurement of contact angle. Any contamination or variation in surface roughness due to the varying cooling rate of the melted wax (during wax specimen preparation) can influence the contact angle [50]. Figure 2 illustrates that the cellulose solution did not cause wetting of any of the three wax surfaces investigated here, as the measured contact angles were > 90°. This high repellency was considered to be a result of the low wettability of wax substances by the cellulose solution. This is due to the low surface energy of the wax and the surface tension of the cellulose solution described by Young's equation. It explains that hydrophobicity can only be observed on solid surfaces with low surface energy [51]. The hydrophobicity of wax and the formation of a high contact angle could result in the formation of microcavities on the cellulose hydrogel surface. Therefore, a mould material creating a lower contact angle is desirable, as this results in higher wettability of the wax mould with cellulose solution. 

**Figure 3:**
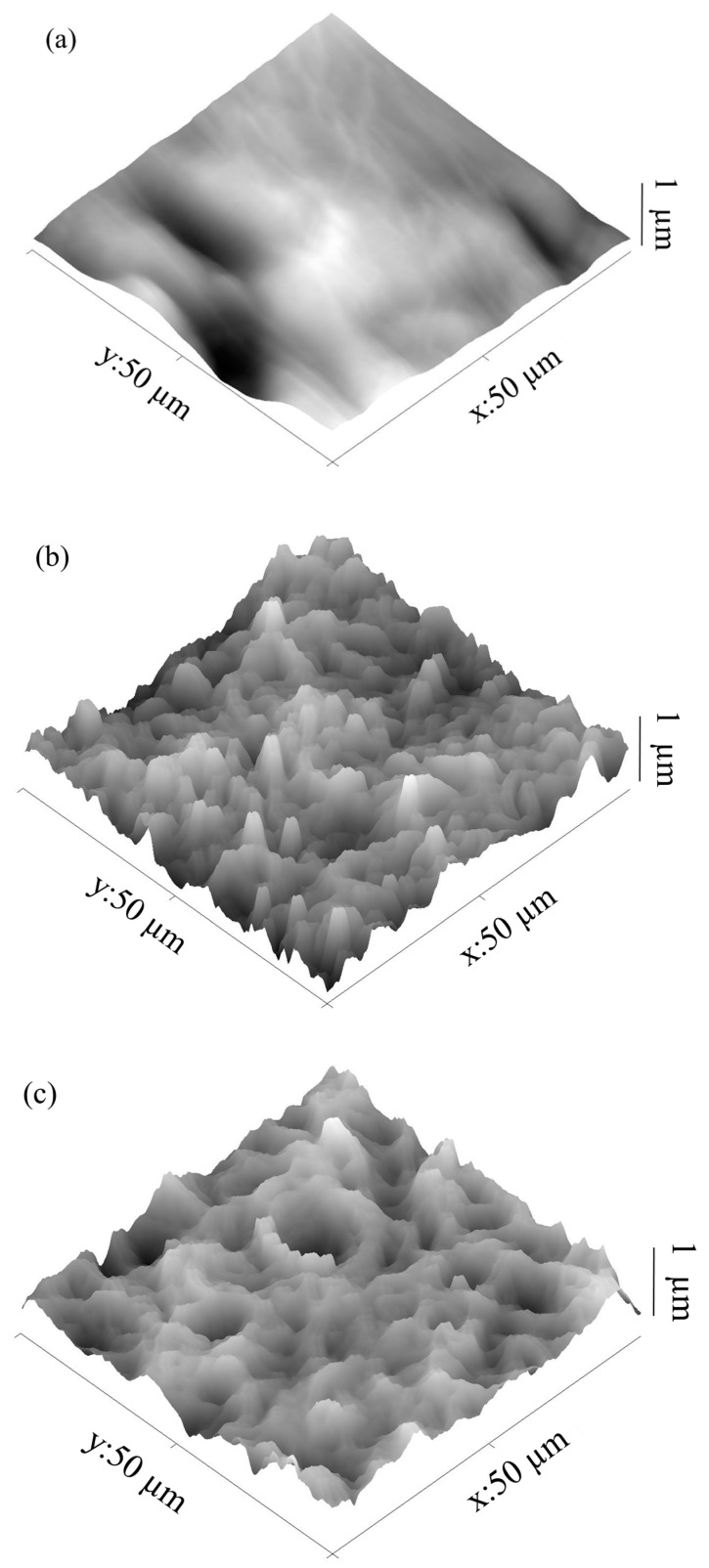
Three-dimensional (3D) atomic force microscopy (AFM) images of waxes in air. (**a**) Paraffin (wax 1) roughness value (Ra): 73 nm, (**b**) sacrificial investment casting wax (wax 2) Ra: 133 nm and (**c**) Solidscape^®^ wax (wax 3) Ra: 117 nm.

The deviation of contact angles among three waxes was assumed to be due to their surface roughness. According to the Cassie and Baxter state [48,52], surface roughness has a major influence on contact angle [50,53,54,55,56]. Increased roughness of a hydrophobic surface leads to a concomitant increase in hydrophobicity; therefore, the surface roughness of the wax moulds was measured. Figure 3 displays AFM images of the prepared wax samples, which reveal the surface roughness. 

The surface of wax 1 appeared wavelike but with a smooth surface compared to waxes 2 and 3 under the AFM (Figure 3). In general, rough surfaces are characterised by one of the two types of wetting, i.e., homogeneous or heterogeneous wetting. In homogeneous wetting, the liquid contacts the surface of a solid and completely fills all grooves. In heterogeneous wetting, the liquid is trapped into grooves, which can be described by the Wenzel relation [48]. The higher microroughness of waxes 2 and 3 relative to wax 1, with its smoother surface, could be the reason for the slight difference in the measured contact angles.

The wettability of cellulose gel by cellulose solution was also studied to understand the possibility of casting cellulose hydrogel through an additive manufacturing process. In this method, the wax mould can be 3D printed with cellulose gel simultaneously [57]. A droplet of the cellulose solution was placed on the cellulose gel and immediately absorbed into the gel, indicating that the polar cellulose solution and cellulose gel were creating hydrogen bonds, including hydrogen bonding between the hydroxyl groups of cellulose gel and cellulose solution molecules (NaOH hydrates, urea hydrates and free water), and also hydrogen bonding and electrostatic interaction between NaOH, urea and water molecules [14,15]. 

### 3.2. Thermophysical Analysis of Moulds

The onset of melting and melting points of the moulds were measured to estimate the gelling temperature of the cellulose solution during the casting process. The phase changes of the investigated waxes were observed over a range of temperatures. The onset of melting temperatures were found to be 57, 47 and 41 °C, and the melting points 64, 58 and 48 °C, for waxes 1, 2 and 3, respectively (Table 1). The DSC thermograms for waxes 1, 2 and 3 are provided in the Supplementary Materials. Previously published thermophysical properties for paraffin (wax 1) indicate similar results [55,56,57]. The increase in melting points among the investigated waxes is a consequence of a change in the composition of waxes, since the melting point of the waxes increases with the increased number of carbon atoms in the wax chain [58].

The gelation of cellulose solution occurs at a temperature range from 10 °C to 65 °C [14]. Moreover, the gelation temperature should not exceed the mould’s onset of melting, to avoid any possible damage to the moulds. Therefore, we set the cellulose gelation temperature for waxes 1, 2 and 3 at 57, 47 and 41 °C, respectively. Considering that the gelation of cellulose solution takes place much more easily at temperatures above 30 °C [14], all three wax mould materials are capable of casting cellulose gel. 

Apart from gelling temperature, other factors in selecting the best wax mould material include mechanical properties, shrinkage and ease of mould removal. The pure form of paraffin wax (wax 1) is brittle and hardly used for any investment casting process. Therefore, it is necessary to modify the crystal properties by adding branching to the existing carbon backbone chain [59]. The branched properties result in a modified paraffin with a higher viscosity, smaller crystalline structure and modified functional properties useful for sacrificial investment casting [60]. Sacrificial wax (wax 2) has a low shrinkage ratio, high hardness and low tackiness [61,62]. Different formulations of sacrificial wax are used for forming a model for precision casting [62]. Solidscape^®^ wax (wax 3) is specifically developed for additive manufacturing [63]. Its enhanced properties, such as low shrinkage ratio and high hardness, make it suitable for investment casting. 

### 3.3. Mould Removal

Mould removal is an important factor in successfully casting cellulose hydrogel. In the proposed method of mould removal, the wax mould with the gelled cellulose part was heated in water. This melted the wax, removing it from the cellulose gel. The melted wax floated to the surface of the water due to the immiscible nature of water and wax. Waxes 1, 2 and 3 (with melting points of 64, 58 and 48 °C, and latent heat of fusion of 186, 113 and 156 J/g) took 3, 2 and 1.5 h, respectively, to remove from the cellulose gel. The variation in mould removal time seems to depend on the different wax melting points, that is, as the mould starts to melt from the outer wax surfaces and separates from the cellulose gel, this makes space for the rest of the wax to melt and remove. Moreover, a lower mould melting point with a low latent heat of fusion for the same amount of wax means a quicker solid-to-liquid phase change with less required energy [64]. Results showed that wax 3, with the lowest melting point of 48 °C and 156 J/g latent heat of fusion, removed faster than the other two moulds.

**Figure 4:**
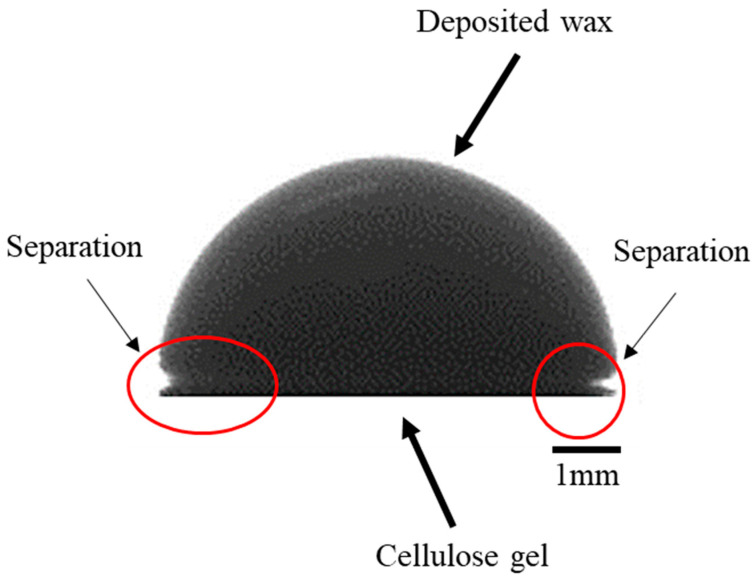
Image of a droplet of melted wax (Solidscape^®^) at 70 °C deposited on cellulose gel.

In addition, we investigated the interaction of melted waxes and cellulose gel by placing droplets of melted wax at 70 °C on the cellulose gel surface. The results indicated that there was no adhesion between all three waxes and the cellulose gel (Figure 4). This is due to the hydrophobicity of wax, as a nonpolar substance, and cellulose gel containing 81 wt. % of water. The melted wax created a high contact angle (>90°) and separated from the cellulose gel surface. Thus, during the mould removal process in hot water at ~90 °C, the wax melted and separated from the cellulose gel. The floating wax could then be recovered from the water and reused. The primary recycling of wax could result in positive economic effects for mass manufacturing cellulose hydrogel over the long term.

**Figure 5:**
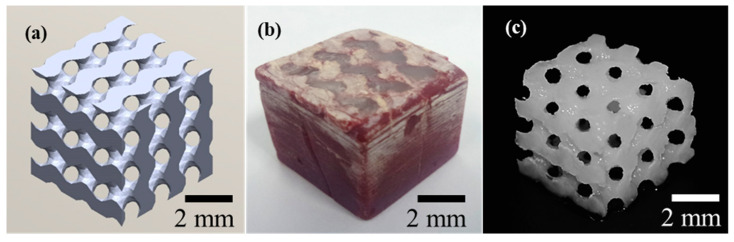
Fabricated cellulose hydrogel with Schoen gyroid structure using Solidscape^®^ wax, channel diameter of 1 mm: (**a**) CAD design of network gyroid with 1-mm channel size, (**b**) gyroid wax mould template filled with cellulose solution and gelled and (**c**) cellulose hydrogel after wax removal.

TPMS cellulose hydrogels were successfully fabricated using the proposed method of sacrificial casting. The results shown in Figure 5 indicate that the casting method allows the fabrication of complex parts with fine features, and thus, confirm the success of the applied method to cast cellulose hydrogel for potential applications, such as tissue engineering and filtration. 

## 4. Conclusions

Wax is a suitable negative template material for the fabrication of cellulose hydrogel using a casting method. In this study, paraffin, lost wax and Solidscape^®^ wax were used to make moulds. The interaction of the cellulose solution and the wax mould was characterised by the mean of the contact angle. Wettability tests showed that the cellulose solution creates a high contact angle with wax moulds, indicating a hydrophobic surface. The deviation between the measured wax contact angles was due to the different surface energy and the microroughness of the moulds’ surfaces. The hydrophobicity of wax moulds is beneficial in mould removal and wax recycling. The difference in the time required to remove the wax can be assumed to be due to the different melting points of the different wax moulds. 

## Figures and Tables

**Table 1:** Melting behaviour of waxes 1, 2 and 3 at various cooling and heating rates.

	Cooling Rate °C/min	Heating Rate °C/min	Melting Temp. °C	Onset of Melting °C	Latent Heat of Fusion J/g	Supplementary Figures
Wax 1	10	10	65.82	57.26	183.7	Figure S1
	25	10	65.72	57.36	186.3	Figure S2
	50	1	64.25	57.88	208.5	Figure S3
Wax 2	10	10	59.66	45.97	115.8	Figure S4
	25	10	58.82	46.43	113.4	Figure S5
	50	1	56.69	48.60	121.4	Figure S6
Wax 3	10	10	48.58	40.79	155.4	Figure S7
	25	10	48.52	41.14	156.7	Figure S8
	50	1	47.77	41.41	212.9	Figure S9

## Supplementary Materials

The following are available online at https://www.mdpi.com/2306-5354/7/2/58/s1, Figure S1: DSC thermogram of wax 1, recorded at a heating rate of 10 °C/min; Figure S2: DSC thermogram of wax 1, recorded at a heating rate of 25 °C/min; Figure S3: DSC thermogram of wax 1, recorded at a heating rate of 1 °C/min; Figure S4: DSC thermogram of wax 2, recorded at a heating rate 10 °C/min; Figure S5: DSC thermogram of wax 2, recorded at a heating rate 25 °C/min; Figure S6: DSC thermogram of wax 2, recorded at a heating rate 1 °C/min; Figure S7: DSC thermogram of wax 3, recorded at a heating rate 10 °C/min; Figure S8: DSC thermogram of wax 3, recorded at a heating rate 25 °C/min; Figure S9: DSC thermogram of wax 3, recorded at a heating rate 1 °C/min. Table S1: Melting behaviour of wax 1, 2 and 3 at various cooling and heating rates.
