# Supplementary material for: Complex Geometry Cellulose Hydrogels Using a Direct Casting Method
Source: Bioengineering (Basel). 2020 Jun 16;7(2):58. doi: 10.3390/bioengineering7020058 (PMC7355674; doi:10.3390/bioengineering7020058)
Supplement: Supplementary file 1 [file bioengineering-07-00058-s001.pdf]

bioengineering

Supplementary materials: Complex geometry cellulose hydrogels using a direct casting method

Hossein Najaf Zadeh, Tim Huber, Volker Nock, Conan Fee, and Don Clucas

Table S1: Melting behaviour of wax 1, 2 and 3 at various cooling and heating rates.

Figure Number	Wax sample	Cooling rate °C/min	Heating rate °C/min	Melting temp. °C
Figure S1	Wax 1	10	10	65.82
Figure S2	Wax 1	25	10	65.72
Figure S3	Wax 1	50	1	64.25
Figure S4	Wax 2	10	10	59.66
Figure S5	Wax 2	25	10	58.82
Figure S6	Wax 2	50	1	56.69
Figure S7	Wax 3	10	10	48.58
Figure S8	Wax 3	25	10	48.52
Figure S9	Wax 3	50	1	47.77

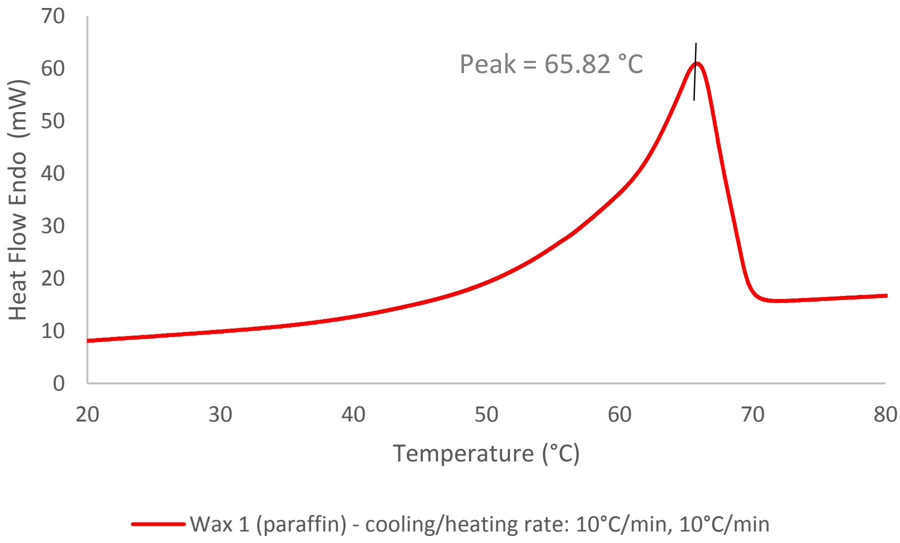

Figure S1: DSC thermogram of wax 1, recorded at a heating rate of 10 °C/min.

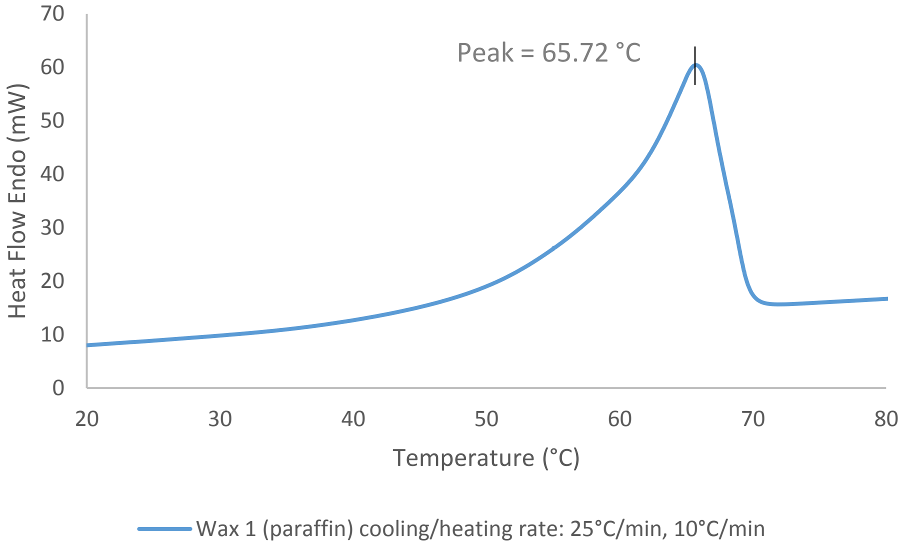

Figure S2: DSC thermogram of wax 1, recorded at a heating rate of 25 °C/min.

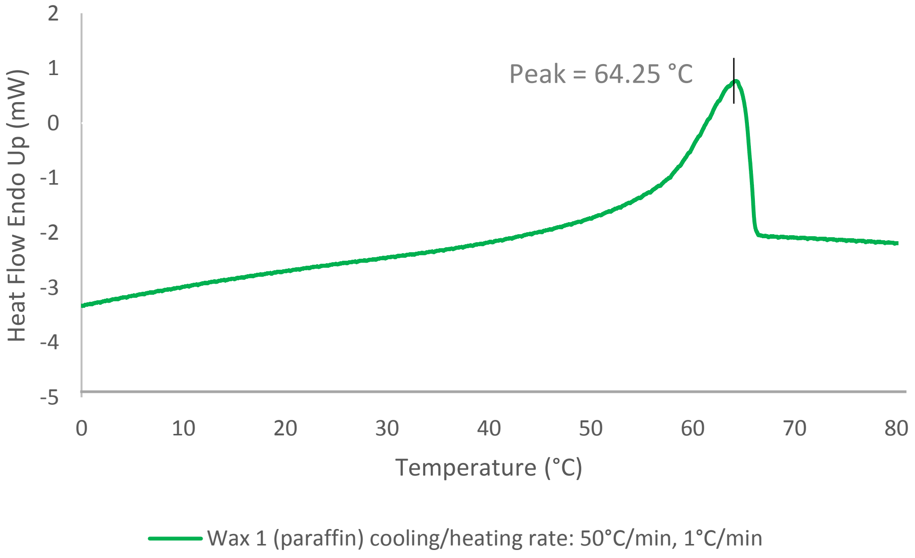

Figure S3: DSC thermogram of wax 1, recorded at a heating rate of 1 °C/min.

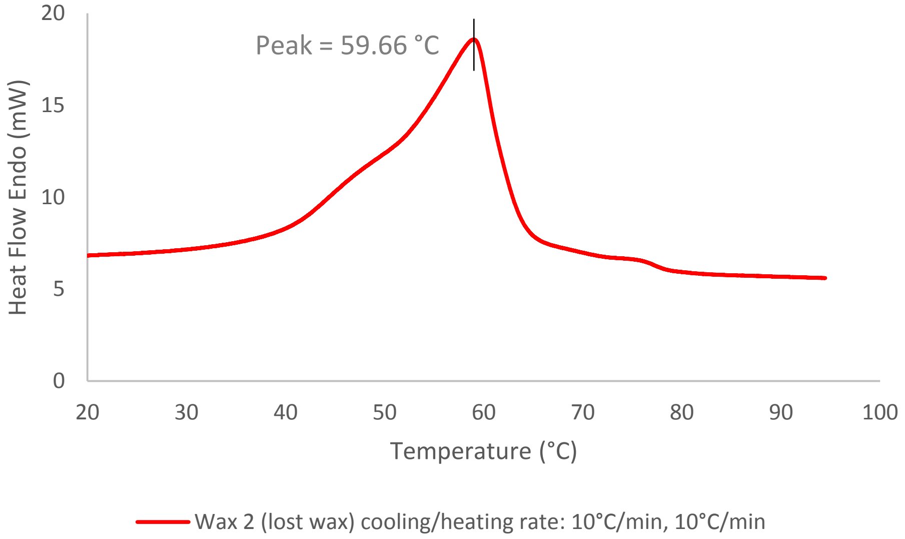

Figure S4: DSC thermogram of wax 2, recorded at a heating rate 10°C/min.

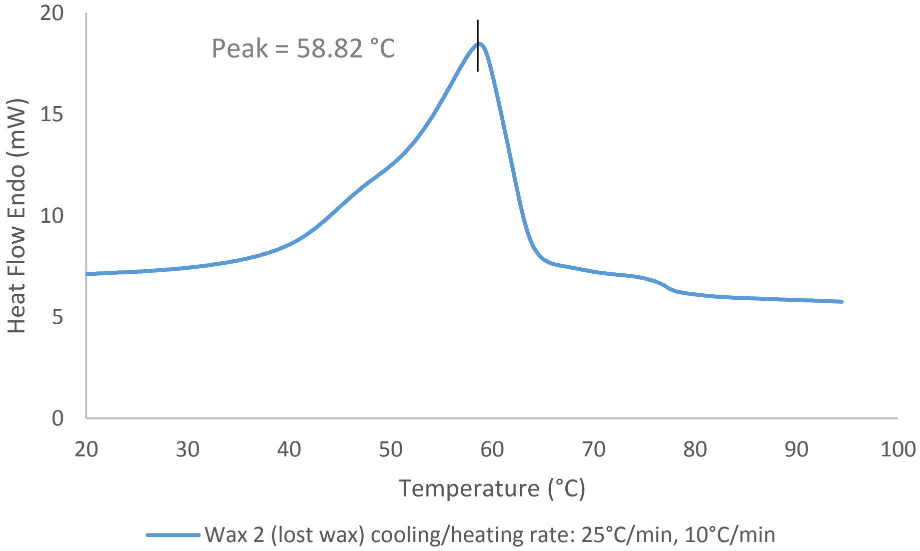

Figure S5: DSC thermogram of wax 2, recorded at a heating rate 25°C/min.

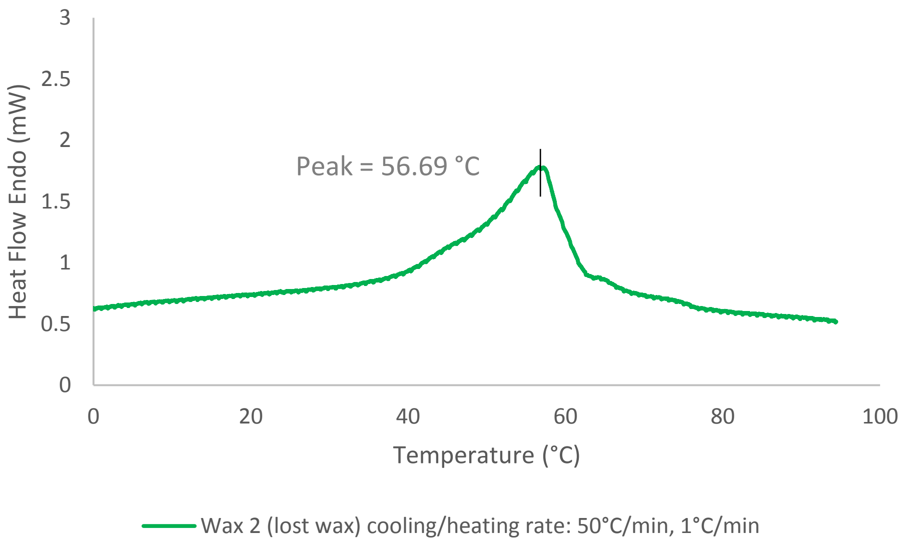

Figure S6: DSC thermogram of wax 2, recorded at a heating rate 1°C/min.

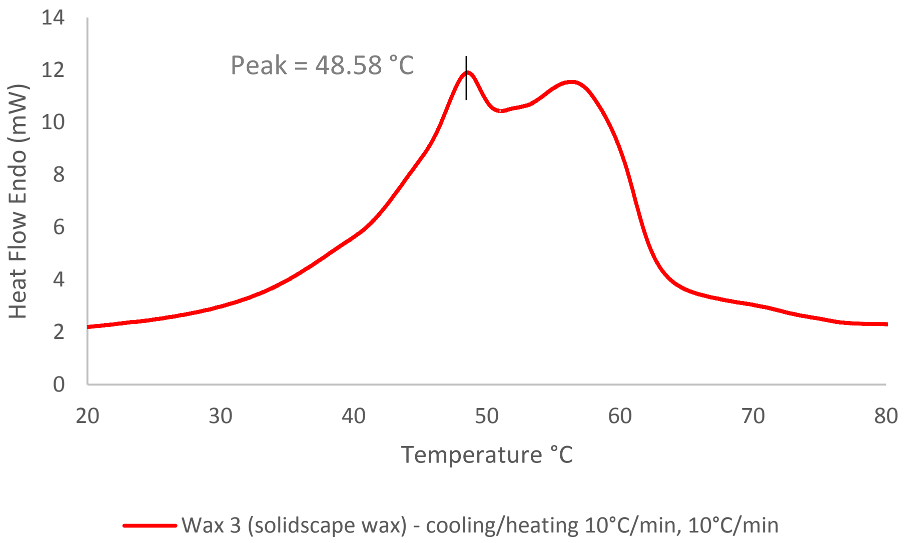

Figure S7: DSC thermogram of wax 3, recorded at a heating rate 10°C/min.

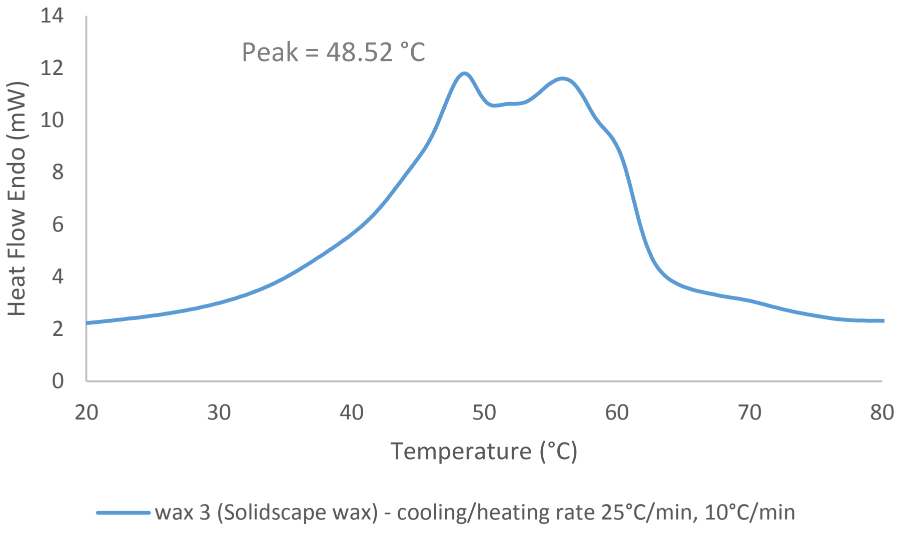

Figure S8: DSC thermogram of wax 3, recorded at a heating rate 25°C/min.

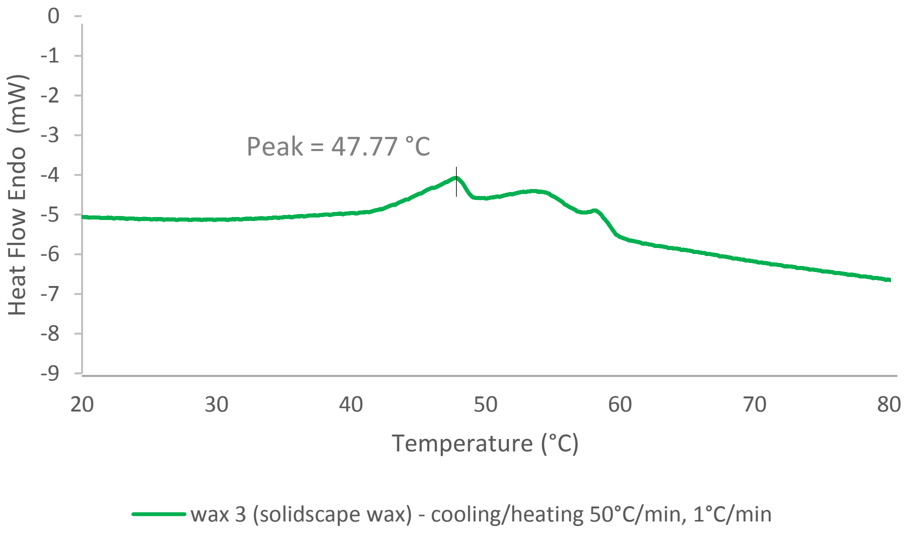

Figure S9: DSC thermogram of wax 3, recorded at a heating rate 1°C/min.
